# A tractable physical model for the yeast polarity predicts epistasis and fitness

**DOI:** 10.1098/rstb.2022.0044

**Published:** 2023-05-22

**Authors:** Werner Karl-Gustav Daalman, Els Sweep, Liedewij Laan

**Affiliations:** Department of Bionanoscience, TU Delft, 2629 HZ Delft, The Netherlands

**Keywords:** genotype–phenotype map, epistasis, budding yeast, polarity, bottom-up modelling

## Abstract

Accurate phenotype prediction based on genetic information has numerous societal applications, such as crop design or cellular factories. Epistasis, when biological components interact, complicates modelling phenotypes from genotypes. Here we show an approach to mitigate this complication for polarity establishment in budding yeast, where mechanistic information is abundant. We coarse-grain molecular interactions into a so-called mesotype, which we combine with gene expression noise into a physical cell cycle model. First, we show with computer simulations that the mesotype allows validation of the most current biochemical polarity models by quantitatively matching doubling times. Second, the mesotype elucidates epistasis emergence as exemplified by evaluating the predicted mutational effect of key polarity protein Bem1p when combined with known interactors or under different growth conditions. This example also illustrates how unlikely evolutionary trajectories can become more accessible. The tractability of our biophysically justifiable approach inspires a road-map towards bottom-up modelling complementary to statistical inferences.

This article is part of the theme issue ‘Interdisciplinary approaches to predicting evolutionary biology’.

## 1. Introduction

Many fields, such as personalized medicine [1], agriculture [2], chemical production [3] and forensics [4], will greatly benefit from better understanding of how traits connect to genes, the so-called genotype–phenotype (GP-) map. However, resolving this connection is generally not straightforward even for known heritable traits [5]. For example, multiple genes can contribute to a single trait (polygenic inheritance) while multiple traits can emerge from a single gene (pleiotropy) [6]. Frequently, mutational effects have been shown to be non-additive in (model) species as *Escherichia coli* [7] and *Saccharomyces cerevisiae* (budding yeast) [8]. This phenomenon is known as epistasis. Theoretically, epistasis surfaces easily based on metabolic network analysis [9], and some molecular origins are known [10]. As epistasis complicates the predictions of phenotype and consequently gene evolution [11,12], it poses an important challenge for GP-map models.

**Figure 1.**
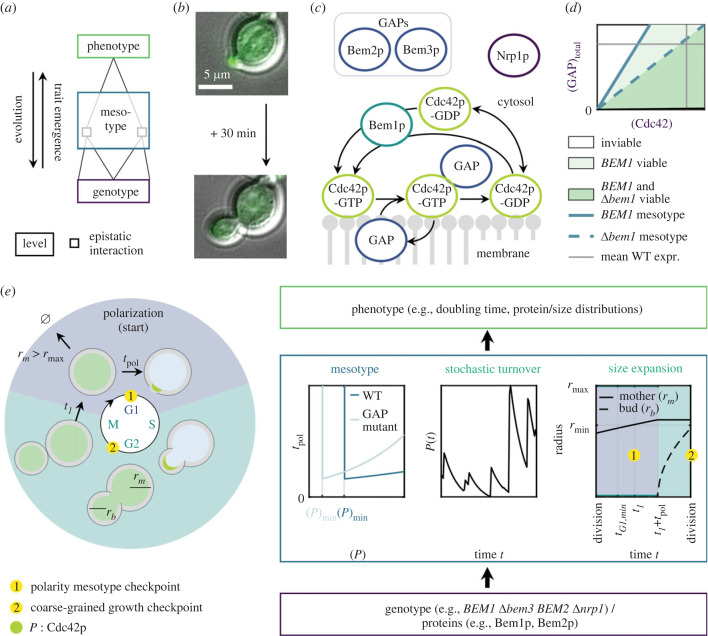
Decomposition of the genotype–phenotype (GP-) map with the mesotype as an intermediate, showcased in yeast polarity. (*a*) General conceptualization of a GP-map containing epistatic interactions. Introduction of an intermediate level simplifies the connections between genes to phenotype. (*b*) Visualization of yeast polarity as the function template for decomposing a GP-map. Example overlay of brightfield and widefield fluorescence images of a polarizing budding yeast cell (yLL129), scale bar 5 µm. Spa2p, binding partner of active Cdc42p, is fluorescently labelled. (*c*) Schematic overview of core polarity protein network (proteins not to scale). Proteins are denoted with a suffix -p, as opposed to italicized gene names. A positive feedback for (active) Cdc42p-GTP is mediated by the Bem1p. Nrp1p represents a mechanistic unknown. (*d*) Phase diagram summarizing the GP-map, depicting the phenotype viability (green) as function of genotype (purple) through Cdc42p (active and inactive) and GAP concentration in the cell with or without Bem1p. An intermediate, the ‘mesotype’, can be identified here as the limiting Cdc42p concentration (blue). (*e*) Implementation of the mesotype into a physical cell cycle model, to tractably decompose the polarity GP-map. Starting from G1, every cell aims to divide once the polarity mesotype checkpoint has passed. This implies mother radius *r_m_* exceeding minimum radius *r*_min_, time thus far in G1 *t_1_* exceeding minimum G1 time *t_G1_*_,min_, and a Cdc42 concentration (abbreviated as (*P*)) exceeding mesotype (*P*)_min_. If maximum size *r*_max_ is exceeded in the process, the cell dies. If the mesotype checkpoint is passed, the mother continues to grow isotropically for polarization time *t*_pol_, which is at least *t*_pol,min_. Then, only the bud grows until the second, coarse-grained growth checkpoint, which involves bud size must be a certain fraction of mother size before division. Throughout all phases, Cdc42p is subject to stochastic protein production and deterministic degradation. Its spatial distributions across the cell cycle are schematically depicted in green inside the cells.

In order to unravel this complication, intermediate levels in the GP-map are commonly employed [13]. An intermediate level is any quantity more complex than individual proteins, but less complex than the phenotype level. This level addition is meant to produce a more modular and hence more tractable GP-map (figure 1*a*). Multiple level examples exist, such as the biofunctional gene ontology level (ontotypes) [14], and the diffuse endophenotypes [15]. Ideally, such a level both facilitates understanding of the emergence of phenotypes from genotypes, and elucidates the handles for evolution, the reverse path in the GP-map. Phenomenological or statistical level formulations have the advantage that predictions can be generated in large quantities, integrating data from high-throughput studies [16]. For example, the ontotype approach [14] is proficient in predicting millions of interactions between genes. While these methods are very effective in predicting, there is room to complement these by an alternative approach and level formulation when we pursue a different goal. Here, we aim to maximize understanding of specific, unusual phenotypes and in particular increase our fundamental understanding of emergence of biological network properties and their evolution.

By bottom-up modelling, we hope to circumvent possible interpretability limitations that may result from phenomenological methods and find GP-map rules justifiable from the bottom-up which permit generalizations to less studied networks. In systems with well-mixed proteins, these models are easily scalable, e.g. greater than 100 protein species in a macrophage polarization model [17], and greater than 1000 for *S. cerevisiae* metabolism [18]. However, in systems as yeast polarity where we cannot neglect the spatio-temporal interplay of protein species, tractability requires us to coarse-grain the underlying biochemical networks. We define the quantity that emerges from the biochemistry as the ‘mesotype’. This mesotype describes the function of a biological module as a function of protein concentration(s), and can for example be a sigmoidal Hill curve. Moreover, if this curve is sufficiently simple as we will see in our case here, we can even reduce the mesotype function by a simple scalar/threshold.

To test our mesotype approach, we model polarity establishment in *S. cerevisiae*, the cell cycle step where budding yeast breaks its spherical symmetry to direct bud growth (figure 1*b*). The challenge is reproducing the ample epistasis exhibited in the polarity network in e.g. doubling times [19]. Conveniently, the molecular interaction network for polarity establishment (simplified in figure 1*c*) was recently modelled in broad agreement with literature [20]. In short, polarity is essential for cell proliferation and relies on the small GTPase Cdc42p [21] (proteins denoted with suffix -p, as opposed to italicized genes). Cdc42p is considered active when bound to a GTP nucleotide and can in this form redirect the actin cytoskeleton [22]. Subsequently, growth becomes polarized (only to one direction) instead of isotropic, and a new cell (bud) is formed (see for a review e.g. [23]). Before this growth transition can occur, active Cdc42p must have been clustered to one point in the plasma membrane through multiple positive feedbacks [24]. One positive feedback is mediated by Bem1p to recruit Cdc42p [25–27]. Elsewhere on the membrane, Cdc42p must be inactivated through hydrolysis of its nucleotide. This hydrolysis is catalysed by the confusingly named GTPase activating proteins (GAPs) such as Bem2p and Bem3p [28]. In the absence of Bem1p, clusters of active Cdc42p can still arise, but this process then becomes very sensitive to GAP concentration. Concretely, at low enough GAP concentration relative to Cdc42p concentration, a spot with large Cdc42p concentration can locally saturate the GAPs causing the active Cdc42p spot to be maintained [20]. This mechanism strongly links viable Cdc42p and GAP concentrations. Consequently, deletion of *BEM1* in an experimental evolution experiment structurally led to subsequent deletion of GAPs to restore fast polarity establishment [19]. However, in this experiment another, mysterious protein surfaced, Nrp1p whose absence becomes strongly beneficial in the Δ*bem1* background. Possibly, Nrp1p influences cell cycle timing, but its molecular mechanism is currently unknown. Nevertheless, we include Nrp1p to test our model with the experimental data in [19].

In our system, the mesotype is based on [20], where the current biochemical network model for polarity was specified in terms of the chemical reactions and diffusion of relevant proteins such as Cdc42p, GAPs and Bem1p complexes. Numerical simulations of the reaction–diffusion equations and mathematical analysis thereof then displayed elegantly simple dependencies of polarization success (and time required) on polarity proteins. In particular, polarization is only possible between a minimal and maximal Cdc42/GAP ratio, with the viable range differing per genotype (figure 1*d*). However, the theoretical upper bound of this ratio is not experimentally relevant for this paper, as high expression of Cdc42p under the Gal1-promoter did not reveal severe fitness effects (even though the model in [20] surprisingly shows that a large excess [Cdc42p] above the mesotype should eventually slow down polarization [20]), nor did deleting two GAPs [19]. Therefore, we coarse-grain the biochemistry underlying polarity to a mesotype defined by a minimum Cdc42p concentration below which polarization is not possible. This simple rule on protein dosage will facilitate the understanding of epistasis emergence while sharply reducing computational costs.

To complete our GP-map model, the mesotype is incorporated into a physical cell cycle model, which is further composed of simple volume growth and stochastic protein production (figure 1*e*), to reproduce phenotypes. The phenotypes we consider are G1 times, cell sizes (electronic supplementary material, figure S5) and fitness/growth rates. The latter also encompasses epistasis as it can be seen as an unexpected double mutant fitness, given the fitness effects of single mutants. In this paper we assess the quality of our predictions by comparing these to documented interactions, validating the underlying molecular polarity model quantitatively. Our tractable approach also illustrates how epistasis and therefore feasible evolutionary trajectories depend on growth conditions. Finally, we show our framework relies on biofunctional (and ideally mechanistic information) of the key proteins to yield informative predictions, which delineates the appropriate conditions for applying our method.

## 2. Results

### (a) Cell cycle model design with the mesotype as level between genotype and phenotype

As polarity relies crucially on polarity protein concentrations [20], an accurate transition of polarity to cellular phenotypes includes the time-dependence of key protein concentrations, which in turn implies describing (stochastic) protein production and dilution effects. Therefore, we modelled the yeast cell cycle as a process involving three modules, namely: (i) polarity mesotype, (ii) stochastic protein turnover, and (iii) cell size expansion (figure 1*e*). We sketch the essence of each module, and further details are found in the electronic supplementary material, Information (Section Extended explanation cell cycle model). Moreover, a summary of model parameter values is given in electronic supplementary material, table S1, where we distinguish five parameters (mesotypes of the Δ*bem1*/*BEM1* background, mesotype effects of a Bem2p/Bem3p deletion and a minimum G1 time effect of the Nrp1p deletion) which we use here as fit parameters on fitness values, and other model parameters that are fixed based on observations from literature or theoretical considerations. We integrated the three modules into a simulated population of cells, with properties size and protein content. We employed a Gillespie-style algorithm [29,30], where [Cdc42p] is updated at each simulation step per cell for production bursts (electronic supplementary material, figure S3*a*). The durations between steps follow from random draws of an exponential distribution. The simulations result in phenotypes such as dosage distribution, size and doubling times. These phenotypes each converge to equilibrium values at the end of the simulations (electronic supplementary material, figure S3*b,c*). The convergence of the doubling times also provides a proxy of the error on the simulated doubling time estimate, which is taken at the end of the simulations, and it is typically small (usually less than 1%; see the electronic supplementary material, Convergence) compared to the experimental error in [19].

**Figure 2.**
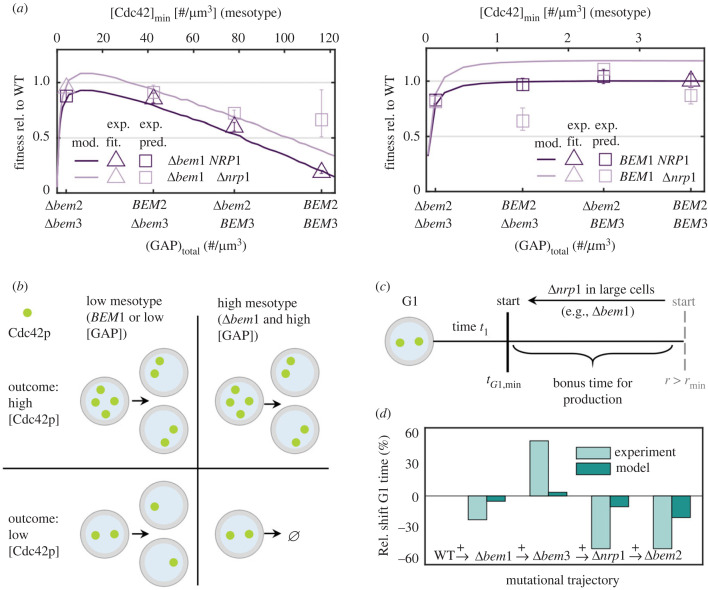
Our physical cell cycle model including the mesotype permits detailed and tractable validation of the underlying biochemical polarity model. (*a*) Experimental fitness values (defined as the reciprocal of population doubling time) relative to wild-type (WT) (phenotypes) for 16 different polarity genotypes [19] denoted by squares when used for validating our physical cell cycle model predictions or by triangles when used for fitting the model. Experimental errors come from the standard deviation of the doubling times across replicates. Model simulations are depicted by the dark and light lines for Δ*nrp1* and *NRP1* background respectively. GAP genotypes link linearly to the minimum Cdc42p concentration to polarize, the mesotype (top axis). Error of the *BEM1 BEM3* Δ*bem2* Δ*nrp1* was not available because of limited data in [19]. As a proxy for the error on the simulation lines, the change in doubling times in the last simulated 100 min. is usually less than 1% and rarely greater than 3% (see the electronic supplementary material, Information, section Convergence), well below the typical experimental error. (*b*) Graphical representation of the effects of GAP (Bem2p and/or Bem3p) and *bem1* deletions from the mesotype perspective. (*c*) Graphical representation of the effects of the *nrp1* deletion from the mesotype perspective. When cells are less fit and become larger, the minimal G1 time reduction associated with the *nrp1* deletion becomes most apparent. (*d*) The detailed phenotype of minimum G1 time as displayed by WT and four polarity mutants, comprising a single evolutionary trajectory. Experimental values are from [19] in light emerald (defined there as time to first polarity spot), model values are in dark emerald (defined here as the time in G1 until both the size and time criteria are met). Each column denoted the relative change in G1 time compared to the previous step in the trajectory.

For module (i), we reduced the biochemical network for polarity to the mesotype of a minimal [Cdc42p] threshold. As depicted in figure 2*d*, the mesotype threshold scales linearly with GAP concentration, here Bem2p and Bem3p concentration, and also depends on Bem1p. Therefore, the mesotypes of all mutants are determined by the slopes of the mesotype lines and crossing points with GAP concentration of the mutant of choice. As the presence of Bem1p increases this slope, we require the slope with and without Bem1p and the (effective) GAP concentration decrease upon Bem2p or Bem3p deletion (thus four parameters in total) to fully describe the mesotype of all mutants of [19]. The mesotype in part constitutes the polarity mesotype checkpoint (see module (iii)). Upon checkpoint passage, cell expansion continues for a time *t*_pol_, which increases exponentially with the excess [Cdc42p]. This time period simplifies the functional dependency uncovered with the aforementioned analysis of the underlying reaction–diffusion equations [20].

**Figure 3.**
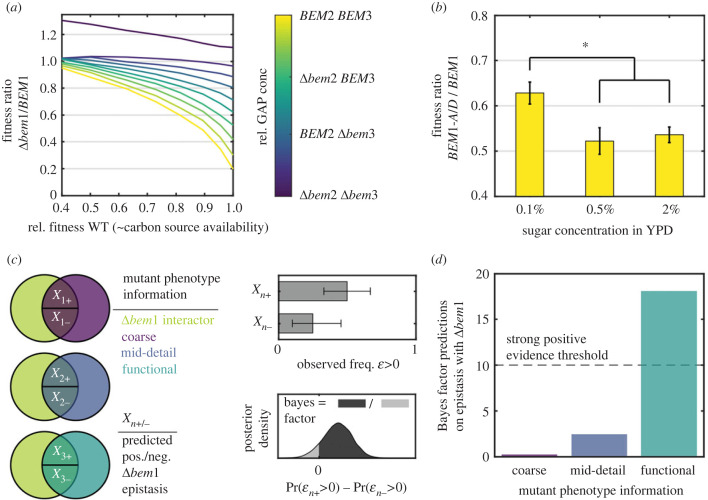
The mesotype allows evolutionary relevant predictions on the effect of carbon source availability and epistasis. (*a*) Simulated fitness differences between *BEM1* and Δ*bem1* backgrounds as a function of carbon source availability, integrated through varying modelled cell membrane area growth rates. Fitness is defined as the reciprocal of population doubling time. Median convergence in doubling time as proxy for the simulation error (see the electronic supplementary material, Information section Convergence) is about 0.5%. The carbon source availability translates to an associated WT fitness. Generally, poorer medium reduces differences in fitness and genetic interactions between GAPs when comparing the *BEM1* and Δ*bem1* backgrounds. (*b*) Experimental assay comparing the effect of the *bem1* deletion in rich and poor medium. Bars denote the fitness plotted of the effective Δ*bem1* background (*BEM1-AID* with added auxin) relative to the *BEM1* background (all with *BEM2* and *BEM3*), for three carbon source availability conditions. Asterisks denote significant differences. (*c*) Workflow for model prediction on epistasis *ε* of various mutants with Δ*bem1*. Data in this panel is illustrative. Mutants are divided into three sets by the intersection of Bem1 interactors with mutants of various phenotype specificity (and hence varying model implementation). Each set consists of two subsets, depending on the model prediction of epistasis sign. For each subset, the beta posterior density of the observed positive epistasis fraction can be constructed (from a binomial likelihood and uniform prior). A 95% credible interval is plotted in the bars. The model prediction given the information per set is that the + subset has more positive epistasis than the − subset. This is assessed by the Bayes factor represented by the ratio between areas right and left of zero, of the posterior density of the difference in positive epistasis abundance in both subsets. (*d*) Bayes factors for the model hypothesis; the ratio between the odds that the model prediction of positive epistasis abundance differences in subsets is true and false. A value above 10 indicates strong positive evidence [32].

Although this analysis assumes a steady concentration state (constant protein concentrations), we use the *t*_pol_ from this analysis in our time-dependent concentration setting. This simplification is valid unless this polarization time is slowed specifically by excessively high [Cdc42p] to above an hour, the typical time scale of concentration fluctuations owing to dilution and production bursts (see the electronic supplementary material, figure S3*a*), Given the optimal polarization time of 5 min [31] and a wild-type (WT) doubling time of 83 min [19], the condition *t*_pol_ greater than 1 h translates roughly to fitness relative to WT of less than 0.6. This threshold typically holds for the experimentally explored genotypes with relatively low mesotypes/excess [Cdc42p], and is marginally crossed for two lowest carbon source availabilities in the Δ*bem3* Δ*bem2* in figure 3*a*.

For module (ii), we only consider Cdc42p to induce cell-to-cell variability, as GAP dosage population noise [33] is much smaller than the noise of Cdc42p (coefficient of variation 0.83, this study). As messenger RNA of Cdc42 lives much shorter than the protein [34,35], we assume bursty Cdc42p production, whose properties can be inferred from flow cytometry experiments (see the electronic supplementary material, figure S1) under these conditions [36]. By contrast, we assume deterministic Cdc42p degradation owing to its high abundance [37].

For module (iii), we assume two stages of constant outer membrane area growth in G1 and S/G2/M phase, respectively. Two stages are a simple description consistent with different phases of growth in [38,39], with alternating mother and bud growth [39], and the constant area growth assumption is not critical (electronic supplementary material, figure S4). In the first stage, spherical mother cells of radius *r*_m_(*t*) grow until the polarity mesotype checkpoint is passed and polarization is completed, or until cell death when *r*_m_(*t*) exceeds maximum radius *r*_max_. The polarity checkpoint entails next to the [Cdc42p]_min_ of module (i), exceeding a minimal radius *r*_min_, and a minimal time since last division *t_G1_*_,min_. The last two criteria reflect the biology behind the Start transition. There, time must be spent to make Ydj1p sufficiently available to release key cyclin Cln3p from the endoplasmic reticulum [40]. The critical size condition, a minimum size a cell must obtain for cell cycle progression events such as bud emergence, has also long been established in literature [41]. The *nrp1* deletion is phenomenologically incorporated by reducing this minimal time as suggested by [19]. In the next stage, a bud with radius *r*_b_(*t*) grows to 70% of the mother volume (the second checkpoint). Then, mother and bud restart the cell cycle independently. Resulting cell expansion rates are in decent agreement with literature [38,39] (see the electronic supplementary material, figure S2).

### (b) The mesotype enables detailed validation of the underlying biochemical model and bottom-up interpretation of phenotype emergence

Our physical cell cycle model encompassing the three modules relies on the validity of the underlying biochemical network model, which was coarse-grained to the mesotype. While the biochemical network model was tested in [20], quantitatively describing phenotypes at higher levels of organization such as fitness were out of reach as cell cycle and population effects were excluded. Our cell cycle model can capture these phenotypes and thus allows more in-depth validation of the biochemical network model.

To this end, we considered 20 previously documented experimental values from [19]. These constitute doubling times, whose reciprocals are defined as fitness, and cell cycle times. The three most prominent, non-trivial phenotypes are (i) strong epistasis in growth rates between GAP (*bem2*/*bem3*) mutants only in the Δ*bem1* background, (ii) strong epistasis between *BEM1* and *NRP1*, and (iii) non-monotonous optimization of G1 times for (reconstructed) experimentally evolved mutants starting from Δ*bem1*. For the latter phenotype, the acceleration of G1 speed of the Δ*bem1* cells, despite their poor fitness, compared to WT cells is particularly noteworthy. While these phenotypes comprise a limited set, these fit the purpose of our model to in particular improve understanding of non-trivial phenotype emergence, rather than generating predictions in bulk. Notwithstanding, more diverse predictions are possible, for example the cell sizes of the aforementioned reconstructed Δ*bem1* background mutants (electronic supplementary material, figure S5).

To model the 16 mutants of [19] in our target observable set, we required five fitting parameters (see also the electronic supplementary material, table S1). First, the *BEM1* and Δ*bem1* have two different values for the mesotype. Second, because the mesotype scales with GAP concentration (figure 1*d*), any combination of *bem2* and *bem3* null mutations, which decrease the total effective GAP concentration, is described by two additional parameters. Last, the *nrp1* mutant is phenomenologically incorporated through a (25%) decrease in *t_G1_*_,min_. To fix these, we first fit these values by considering doubling times of five genotypes [19], a set which covers high and low fitness genotypes, namely those of WT, Δ*bem1*, Δ*bem1*Δ*bem3*, Δ*bem1*Δ*bem2* and Δ*bem1*Δ*bem3*Δ*bem2*Δ*nrp1*. The remaining 11 doubling times can then be used to validate model predictions. In the electronic supplementary material, figure S6 we tested other genotype sets for fitting, all with quantitatively almost identical results and the same conclusions. The fitted parameter values are 116.2 Cdc42p proteins/µm^3^ (Δ*bem1* mesotype), 3.7 Cdc42p proteins/µm^3^ (*BEM1* mesotype), and those mesotypes decrease with GAP deletions by −64% (Δ*bem3*) and −33% (Δ*bem2*).

When considering the first of our targeted phenotypes, GAP epistasis, we see it is quantitatively reasonably described (figure 2*a*, left). Here, the Bem2p and/or Bem3p deletion mutants reside on points along the GAP concentration axis (as in figure 3*a*), with the exact location depending on the model fit. This description is robust to several model assumption modifications (electronic supplementary material, figure S4). Particularly in presence of *NRP1*, fitness values were modelled in accurate accordance with experiments of [19]. As expected, less cells produce sufficient Cdc42p for high mesotypes, leading to increased cell death and lower fitness (figure 2*b*). Therefore, we see a diffuse viability threshold despite a sharp mesotype, as in previous experiments with inducible Cdc42p [20].

For the second phenotype of interest, *BEM1*-*NRP1* epistasis, we see fitness values of the Δ*nrp1* background (figure 2*a*, right) are not always well fitted (4 out of 7 correct within experimental error), although these have relatively large experimental uncertainties. Nevertheless, the strongest feature, the *BEM1*-*NRP1* epistasis, is at least qualitatively described. This description confirms the intuition offered by the mesotype. If the *nrp1* deletion reduces the mandatory G1 waiting time *t_G1_*_,min_, Δ*bem1* cells have more chance to exploit temporary Cdc42p overproduction before excessive dilution, thereby improving fitness (figure 2*c*). Quantitatively, we explain one-third of the epistasis following the definition of [42]. An alternative G1 time formulation (see the electronic supplementary material, table S2) does not strongly alter this result. As a likely reason for the incomplete description, we confirm in the next section that incorporating mutants phenomenologically rather than based on molecular information limits the accuracy of phenotype description.

Last, we turned to the third phenotype of interest concerning non-monotonous G1-time shifts during adaptation. These shifts are an example of the more detailed traits that can be modelled. As the underlying experimental data of [19] were performed in suboptimal (for growth) synthetic medium for fluorescence microscopy, we must account for this slower growth medium in our model. For this purpose, we performed the simulations with half the normal membrane area rates which slows the WT doubling time from 83 min to 105 min. The trends presented here are not very sensitive to the precise membrane growth rate choice (see the electronic supplementary material, figure S7). The observed trends in G1 times along the evolutionary trajectory from WT to the fully evolved mutant in that paper were qualitatively matched, including the unusual G1 time decrease for the Δ*bem1* (figure 2*d*). The mesotype clarifies this subtle phenotype. The Δ*bem1* cells are relatively larger and less limited by the minimum size criterion *r*_min_. This eliminates a potential waiting step in G1 for this background, which may allow the cells to pass G1 faster if these have the stochastic outcome of Cdc42p overproduction. Other phases are on average extended by the relatively high minimum Cdc42 concentration threshold.

In summary, the three phenotype examples illustrate two main points. First, comparison of experimental data with simulation of our physical cell cycle model further confirms the current biochemical model view of polarity. Second, the mesotype framework exhibits the tractability needed for understanding phenotype emergence, as demonstrated with several examples.

### (c) The mesotype generates model predictions for genetic interactions

#### (i) Poorer carbon source availability reduces fitness differences

After establishing the value of the mesotype in trait generation, we turn to the reverse transition in the GP-map. This transition is embodied by evolution (figure 1*a*), since phenotypes such as fitness determine the selective pressure to shape genotypes. Environmental factors are important for growth, with even subtle changes noticeable under highly controlled laboratory settings [43]. Given that historical evolution has occurred in the wild, where conditions are expected to be much more variable and more often difficult than not, adaptive trajectories can be very different than for laboratory evolution. For example, Bem1p is an important yet dispensable part of the polarity network over the fungal tree of life [44], and even a well-functioning budding yeast polarity network without Bem1p is retrieved within a few mutational steps [19], which however do not always improve fitness individually. One may ask how relevant this retrieval is given that under rich laboratory conditions the loss of Bem1p leads to a large fitness loss. One answer to this question is that slower growth conditions and concordant ease of exceeding the mesotype, may make the loss of the protein Bem1 more likely than initially anticipated.

To further substantiate our mesotype intuition on condition-dependence for evolving a network without Bem1p, we first simulate the effect of changes in carbon source richness in the growth media through a change in membrane area growth rates. We considered a roughly three-fold area growth rate range that caused WT fitness to span between 0.5 and 1 (normalized to maximum growth). We further assume Cdc42p expression remains the same across media, which is at least true upon switching from dextrose to ethanol, an inferior carbon source [45].

As a result of our model simulations, figure 3*a* displays the fitness ratio between the Δ*bem1* and *BEM1* background, as a function of GAP concentration (ranging from Δ*bem3* Δ*bem2* to *BEM3 BEM2*) and carbon source availability. Intuitively, we expected Δ*bem1* cells to benefit greatly from less Cdc42p dilution and the extra time to exceed the mesotype threshold. We indeed observe the trend of smaller fitness differences for decreasing GAP concentrations and decreasing carbon source availability in our simulations. This observation suggests poorer media acts as a fitness equalizer, facilitating the evolution of Bem1p.

To experimentally test our hypothesized environmental effect, figure 3*b* demonstrates the effect of poorer medium on fitness with and without Bem1p. In this case, we modulated carbon source availability through sugar content (from 2% to 0.1%). To avoid lengthy exposure of the Δ*bem1* background to high selective pressure, this genotype is mimicked by auxin-inducible degradation [46] of Bem1p. As it is difficult to exactly integrate the media conditions into the simulations, the match cannot be expected to be quantitative. However, the nullifying effect of poor media on the fitness differences between the Δ*bem1* and *BEM1* backgrounds is visible. The relative fitness compared to WT for the effective Δ*bem1* background in the ‘poorest (in terms of carbon)’ media (0.1% dextrose) is significantly better compared to the ‘richest’ and intermediate medium conditions at 2% and 0.5% dextrose (one-sided Welch's *t*-test, *p*-value 1.3 × 10^−3^ and 3.3 × 10^−3^, respectively), also when considering the Holm-Bonferroni correction [47]. Thus, slower growth medium conditions mitigate to some extent fitness differences and show how the mesotype framework can provide a new, intuitive explanation of otherwise non-trivial environmental interactions that are relevant for evolution.

#### (ii) The polarity mesotype predictions on epistasis become useful when functions of mutated genes are known

While we showed that our cell cycle model produced accurate epistasis predictions, the extensive mechanistic information we used is not always available for other functional modules, such as the TORC1 signalling module involving rapamycin [48,49]. This raises the question about the extendibility of the model predictions beyond polarity. We, therefore, determined the minimal information content needed about yeast mutations to make qualitatively useful epistatic predictions between two genes, when only one gene is inside the polarity network.

For this purpose, we considered high-throughput data on *BEM1* interactors. We categorize those interactors by their individual knockout phenotype where available, yielding three sets (*X_n_* with *n* = 1,2,3) of varying phenotype specificities, i.e. information content with regards to biochemical and biofunctional detail of the phenotype (figure 3*c*, and for more detail see the electronic supplementary material, Information, section Predictions using literature data, and table S7). The coarse phenotype set covers (competitive/fermentative) fitness mutants (1), the mid-detail set (2) G1 mutants (in size/speed), and the functional set (3) covers proteasomal, phospholipid or ribosomal mutants, all of which also interact with *BEM1*. As the phenotypes in set 3 point more precisely to specific modules/functions than set 2, and much more than set 1, set 3 has the highest information content and set 1 the lowest. Each set is then split into two subsets according to the sign of the epistasis predicted by the model (*X_n+_* and *X_n−_* for positive and negative epistasis respectively) for each mutant in the set. These predictions can be intuitively constructed as explained in the next paragraphs or derived from simulations (electronic supplementary material, table S3). We then determined for which set, and thus information content, our model predictions help explain the prevalence of the sign of the observed epistasis (*ε_n+_* and *ε_n+_* for each subset) (see Material and Methods and the electronic supplementary material).

Firstly, we considered the coarse phenotype mutant set. The model predictions for *BEM1* interactors in this set follow from incorporating the single mutant phenotype into an adjusted membrane area growth rate *C*_2_. As seen by the media effect in figure 3*a*, smaller rates mitigate the Δ*bem1* effect. The model prediction is hence that deleterious mutants, which slow down growth, are expected to have more positive epistasis with the Δ*bem1* (making this subset *X_1+_*), than beneficial mutants (making this subset *X_1−_*).

To assess this statement, we calculate the (posterior) probability distributions for encountering positive epistasis in literature for subsets *X_1+_* and *X_1−_*, where the inferred probabilities for positive epistasis are defined as *ε_1+_* and *ε_1−_*, respectively. The posterior distributions of *ε_1+_* and *ε_1−_* resulted from assuming a binomial likelihood of finding the observed number of positive interactors *k_1_*_+_ and *k_1−_* from the subsets *X_1+_* and *X_1−_* respectively (*p*(*k_1_*_+_ | *ε_1+_*) and *p*(*k_1−_* | *ε_1−_*)) and an uniform prior for *p*(*ε_1+_*) and *p*(*ε_1−_*) (see also see the electronic supplementary material, Information, section Predictions using literature data). Our statement then mathematically equates to assessing how likely Pr(*ε_1+_* > 0) > Pr(*ε_1−_* > 0) is. We use the Bayesian odds ratio as our metric to set the threshold for sufficient confidence in our statement. This choice involves the probability of Pr(*ε_1+_* > 0) > Pr(*ε_1−_* > 0) divided by its complement, a ratio known as the Bayes factor, to exceed 10 [32]. As seen in figure 3*d*, the model predictions are far from this target, implying our model is not useful to make epistasis predictions based on coarse knock-out growth rate data.

Analogously, we analyse our model predictions for the mid-detail mutant phenotype set. To generate model predictions, the mid-detail mutants are incorporated by changing G1 waiting time *t_G1_*_,min_ (for G1 speed mutants) and additionally the minimal radius before Start *r*_min_ (for G1 size mutants). As was the case for to Nrp1p (figure 2*c*), mutations which allow an earlier entry to Start, disproportionally benefit the Δ*bem1* cells. Therefore, the model prediction is that mutants fast or small in G1 have more positive epistasis with Δ*bem1* than mutants that are slow or large in G1. With a Bayes factor of 2.4, the information content in this set is still insufficient.

Last, we consider the functional mutant set, where model incorporation follows from increasing the Cdc42p half-life for proteasomal mutants, decreasing membrane growth rate *C*_2_ for phospholipid mutants and decreasing the average Cdc42p burst size for ribosomal mutants. The proteasomal and phospholipid mutants mitigate the problematic lack of Cdc42p in the Δ*bem1* cells. Our statement for this set is hence that these two mutant types should therefore exhibit more positive epistasis than the ribosomal mutants, which lowers [Cdc42p]. From the literature data, 29% of the proteasomal and phospholipid mutants have positive epistasis, much more than the 11% of the ribosomal mutants. This results in a Bayes factor of 18, and implies strong positive evidence for our epistasis prevalence statement in this set. This shows we minimally require functional information on mutants for meaningful epistasis predictions, once we have a core where mechanical information is known.

## 3. Discussion

Epistasis forms a general hurdle for reverse-engineering the GP-map, complicating modelling of protein networks and in turn limiting the predictability of phenotypes and evolution. Complementing methods effective to make bulk predictions, we aim to extract understanding and intuition form our predictions. For this purpose and to alleviate the GP-map complexity, we tested the mesotype as an intermediate level between genotype and phenotype. This level emerges from coarse-graining the biophysics of the underlying biochemical network, which in our case corresponded to budding yeast polarity (figure 1). We employed the mesotype, which in our context is the minimum [Cdc42p] to polarize, in a tractable cell cycle model with simple volume growth and stochastic protein production, making bottom-up reconstruction of phenotypes and epistasis feasible and insightful.

First, our cell cycle model allows us to quantitatively confirm that observed population phenotypes are consistent with the existing biochemical network model, which had previously been qualitatively validated [20]. The quantitative validation further increases our trust in the mesotype being a key property of the system. Good quantitative agreement with literature was found for 8 out of 11 polarity mutant doubling times used for predictions, and qualitative agreement for documented unintuitive G1 times (figure 2). Phenomenological linkage of minimum G1 time to Nrp1p, a protein normally not included in polarity models, also provided fruitful model predictions. Thus, complementary to big data approaches which may yield superior quantitative matches with phenotype observations, our cell cycle model adds value by generating tractable and interpretable predictions, which also provides direction to deciphering the remaining mechanistic unknowns in the polarity network.

Second, we showed how the mesotype elucidates epistasis. We hypothesize that less carbon availability extends protein production time, alleviating minimum protein concentration thresholds. We verified this for yeast polarity, where the [Cdc42p] mesotype threshold proves less problematic for the Δ*bem1* background in poorer media in simulations and experiments (figure 3*a,b*). The Δ*bem1* case fits the more general picture that haploin sufficiency in YPD (10 g l^−1^ yeast extract, 20 g l^−1^ peptone, 20 g l^−1^ dextrose) is typically lifted in poorer medium [50]. By the same token, evolvability of (near-)essential genes may be enhanced under slow growth conditions, where fitness values across genetic backgrounds converge. In our example, step-wise evolution of Bem1p is still feasible given laboratory conditions [20]. Yet, for other proteins, medium change may be the only manner to circumvent fitness valleys. Ideally, we also explore other environments. For example, we can conjecture that nitrogen limitation changes protein degradation [51], but to what extent this applies to Cdc42p specifically or whether more parameter changes are induced is not clear. However, with more information on the effect of the environment on model parameters, we would be able to assess the effect of other slow growth conditions.

Finally, we determined the basis for successful epistasis predictions. Given the robustness of our results to many alternative growth detail formulations (see the electronic supplementary material, figure S4), we argue the core of our model consist only of dosage noise in combination with a known mesotype. Then, to generate meaningful predictions for epistasis with genes outside the scope of the mesotype, adding functional information for those genes proved necessary and sufficient from examining high-throughput literature data (figure 3*d*), in line with the ontotype strategy [14]. While a sharp mesotype as in polarity implies essentiality (around 19% of yeast genes [52]) or toxicity, generally other mesotypes are more appropriate. Yet, given the simple functions with which fitness landscapes as function of single genes can be fitted [53], the mesotype approach still seems feasible to model many other biological networks.

Using our findings on yeast polarity as a template, we envision a road-map to apply to general GP-maps (electronic supplementary material, figure S8). The core functional component, in this case, polarity, is to be modelled by justifiable coarse-graining, which results in the mesotype of the system. This mesotype in turn emerges from functional subunits [20], identifiable from the rigorous analysis of the underlying biophysics. Currently, yeast polarity stands alone at the ideal intersection between complexity and required mechanistic knowledge. Mesotype generation for other systems would again require detailed numerical analysis of reaction–diffusion systems, or results more simply from dose–reponse curves when spatial information is not essential for the chemical reactions. In the latter case, we essentially revert to a more standard biochemical model, but it is in the former case where spatio-temporal information is essential that the mesotype coarse-graining is key to retain tractability in phenotype predictions. Once multiple model systems with important spatio-temporal dynamics (such as the PAR protein system in *Caenorhabditis elegans* [54], with promising recent advances [55]) have been described in this manner, it may be possible to construct a limited library of recurring subunits. Based on this library, construction of the corresponding mesotype may be much simpler. Moreover, given the possibilities of epistasis predictions beyond polarity given one mesotype, it may become possible to tractably model the full protein network of an organism once all core modules in an organism are captured by mesotypes. This may enable detailed epistasis predictions on diverse kinds of phenotypes, paving the general path to bottom-up (population) phenotype prediction.

## 4. Material and methods

### (a) Strain construction

All strains used in this study are *S. cerevisiae* strains in the W303 background (see the electronic supplementary material, table S9). The *ade2* deletion in yLIC132 was performed through transformation of yLL3a by homologous recombination using the *URA3* marker (polymerase chain reactions (PCRs) using primers olic24, olic15, olic18 and olic26 on a template derived from the pRL368 plasmid [56]). The *URA3* was removed by overlap extension PCR (primers olic24, olic20, olic26 and olic21) to yield yLIC133 selecting on 5-FOA (Zymo Research). In this strain, *osTIR1-KANMX4* was integrated into the HO locus (from plasmid pOsTir1w/oGFP) and *BEM1* was replaced by *BEM1-mCherry-AID* to yield yWT03 by selecting transformants with G418 (Thermo Fisher) and Hygromicin (Formedium). Plasmid pOsTIR1w/oGFP was a gift from Matthias Heinemann (Addgene plasmid no. 102883; http://n2t.net/addgene:102883; RRID:Addgene_102883, while the PCR product for *BEM1-mCherry-AID* is from Addgene plasmid no. 173925; http://n2t.net/addgene:173925; RRID:Addgene_173925). The latter resulted from Gibson assembly of a *BEM1* homology region added to a pG23A plasmid backbone, upstream and in frame of the *mCherry-AID* sequence. Plasmid pG23A was a gift from Matthias Heinemann (Addgene plasmid no. 102884; http://n2t.net/addgene:102884; RRID:Addgene_102884). Further downstream, the *HPHMX6* cassette and another *BEM1* homology region was added. For primers and plasmids see the electronic supplementary material, tables S8 and S10, respectively.

### (b) Fluorescence measurements

Fluorescence data of strains RWS116 [57] and RWS1421 [24] were acquired using FlowJo CE software and performed on a BD FACScan flow cytometer. Cells were pregrown in YNB (0.69% w/v, Sigma-Aldrich) + CSM -Met (0.77% w/v, Formedium) + 2% dextrose (Sigma-Aldrich), diluted to an optical density (OD)_600_ of 0.1 and measured after 15 h. Gamma fits on gated flow cytometry data using maximum likelihood through Matlab's R2016a built-in *mle* function, were done on the background (RWS116) and GFP-Cdc42p data (RWS1421). We subtracted the background by approximating the deconvolved Cdc42 distribution as a gamma distribution [58], whose parameters link to expression burst parameters [36]. For fluorescence microscopy of yLL129a (figure 1*b*), we used low-fluorescence media, namely non-fluorescent nitrogen base (0.69% w/v, Formedium), CSM amino acid mix (0.79% w/v, Formedium) and 2% dextrose (Sigma-Aldrich).

### (c) Growth assays

The growth rate assay for RWS1421 was performed in the same media type as the flow cytometry, in an Infinite M-200 Pro Tecan plate reader at 30°C. OD_600_ measurements (9 nm bandwidth) were conducted with an interval of 12 min for a total duration of 24 h, using a Thermo Fisher Scientific Nunclon 96 Flat Bottom Plate input template. After an initial 1000 s of orbital shaking (1 mm amplitude), linear shaking between measurements (25 flashes, 5 ms settle time) lasted 330 s each time (amplitude 1 mm). OD_600_ values were analysed using a home-written Matlab GUI already used in [20] and made available as the electronic supplementary material, Information. A weighted least-squares (WLS) regression (e.g. [59]) is done on the log (base 2) of the (OD_600_ – background OD_600_ value), where the background is set as the mean of the first 10 OD_600_ values in that well. The weights for WLS are set by the reciprocal of the difference between each log value +/- the instrument error. The instrument error is estimated by taking the standard deviation of the first 10 OD_600_ or if this is zero, by 10^−digit^/2 where digit is the value of the exponent with base 10 of the last significant digit. Within a user-defined bandwidth a fit window moves across the longest time span with data above a user-defined signal-to-noise ratio (with noise from the instrument error). We choose the steepest slope, which corresponds to the fastest doubling time, which pertains to a fit with a *R*^2^ squared above a user-defined value. For this assay, late log phase reflects the flow cytometry conditions the most, so we fit above an OD above 0.1, a fitting window size to 21 points, a minimum *R*^2^ to 0.9 and minimal signal-to-noise ratio to 2.

For the growth rate assay in figure 3*b*, we used YPD (10 g l^−1^ yeast extract, 20 g l^−1^ peptone, 0.1/0.5/2% dextrose, 20 µg ml^−1^ adenine (all Sigma-Aldrich)) and 0.25 mM auxin (Merck Millipore). Strains were then grown in a turning wheel for at least 24 h at 30°C. After this pre-growth the cultures were diluted (at least 100×) to an OD_600_ of approximately. 0.05 into a 96-well plate containing 100 µl medium well^−1^. The OD_600_ values were measured for 48 h using a Biotek Epoch 2 Microplate Spectrophotometer using linear and orbital shaking at 30°C, as described in [20]. Three experiments with two plates each were done, using one and two biological replicates for the *BEM1* and *BEM1-AID* background respectively, with at least two technical replicates per medium. Per run, we averaged the fitness (defined by the reciprocal of the doubling time) of *BEM1* replicates. We then divided the fitness of each *BEM1-AID* strain by the mean *BEM1* fitness of that run. We then pooled all BEM1-AID replicates per medium for data analysis. Wells where no growth were observed were excluded, and we neglected the error from the doubling time fits as the largest error source is the variation across replicates. We used a one-sided Welch *t*-test (Matlab R2016a's native *t*-test2, for unequal variances) to test for significant differences, and applied a Holm-Bonferroni correction factor [47] to the significance values for the three *t*-tests performed.

### (d) Computational

An initial population of 200 cells with 2.4 µm and zero Cdc42 concentration are asynchronized across a bandwidth of 83 min and iteratively grown until a population size value of 2 million. We calculate the doubling times for 67 mesotypes (ranging roughly from 0.026 to 169 Cdc42 proteins µm^−^^3^), both for the *BEM1* and Δ*bem1* background, which differ in *φ*_pol_ (see the electronic supplementary material, table S1). The doubling times of WT, Δ*bem1*, Δ*bem1* Δ*bem3*, Δ*bem1* Δ*bem2* and Δ*bem1* Δ*bem3* Δ*bem2* Δ*nrp1* of [19] were used for fitting in figure 2*a* by minimizing a normalized score objective as function of mesotype, using Matlab R2016a's native *fminsearch*, and by manual inspection for setting *t*_mut_ (to 0.75) for the *nrp1* deletion. This objective is the sum of ((fitted doubling times – observed doubling times)/error of observed doubling times)^2^. Doubling times corresponding to an arbitrary mesotype can then be approximated by interpolation.

Interaction and phenotype data for figure 3*d* were obtained from BioGRID [60] and SGD [61] respectively (date of access 6/8 March 2018), see also the electronic supplementary material, table S6 for detailed references per interaction. We calculated posterior distributions for the probability *ε* of a positive interaction with *BEM1*, using the binomial likelihood for such an interaction in literature and a uniform prior. The posterior distribution for the probability of a positive interaction *ε* is a beta distribution with parameters *k* and *N*, where *k* is the encountered positive interactions and *N* the number of encountered negative interactions with *BEM1* [62]. The positive and negative interaction counts for this study can be found in the electronic supplementary material, table S7.

From 10000 random draws of every *ε*, we can simulate the probability distributions of the differences between phenotype subset pairs of *ε*. Alternative, we can rewrite this probability as a Bayes factor for the model hypotheses that the positive epistasis is more prevalent in the + subset than in the subset.

## Data Availability

We attached the data in a folder as the electronic supplementary material [63].
